# Combined effects of O_3_ and UV radiation on secondary metabolites and endogenous hormones of soybean leaves

**DOI:** 10.1371/journal.pone.0183147

**Published:** 2017-08-14

**Authors:** Bing Mao, Hong Yin, Yan Wang, Tian-Hong Zhao, Rong-Rong Tian, Wei Wang, Jia-Shu Ye

**Affiliations:** 1 Postdoctoral Research Station of Crop Science, College of Agronomy, Shenyang Agricultural University, Shenyang, China; 2 College of Agronomy, Shenyang Agricultural University, Shenyang, China; 3 National Field Observation and Research Station of Shenyang Agro-ecosystems, Institute of Applied Ecology, Chinese Academy of Sciences, Shenyang, China; Nanjing Agricultural University, CHINA

## Abstract

Enhanced ultraviolet radiation (UV) and elevated tropospheric ozone (O_3_) may individually cause reductions in the growth and productivity of important agricultural crops. However, research regarding their combined effects on important agricultural crops is still scarce, especially on changes in secondary metabolites and endogenous hormones, which are important protective substances and signal components that control plant responses to environment stresses. In this study, using an experimental setup of open top chambers, we monitored the responses of seed yield per plant, leaf secondary metabolites and leaf endogenous hormones under the stress of elevated O_3_ and enhanced UV radiation individually, as well as their combined stress. The results indicated that elevated O_3_ (110 ± 10 nmol mol^-1^ for 8 hours per day) and enhanced UV radiation (1.73 kJ h^-1^ m^-2^) significantly decreased seed yield per plant. Concentrations of rutin, queretin and total flavonoids were significantly increased under the elevated O_3_ treatment or the enhanced UV radiation treatment or the combination treatment at flowering and podding stages, and concentrations of rutin, queretin and total flavonoids showed significant correlations with seed yield per plant. Concentrations of ABA and IAA decreased under the three treatments. There was a significant positive correlation between the ABA concentration and seed yield and a negative correlation between the IAA concentration and seed yield. We concluded that the combined stress of elevated O_3_ and UV radiation significantly decreased seed yield per plant. Yield reduction was associated with changes in the concentrations of flavonoids, ABA and IAA in soybean leaves. The effects of the combined O_3_ and UV stress were always greater than those of the individual stresses alone.

## Introduction

Elevated tropospheric O_3_ is one of the most phytotoxic air pollutants that can reduce growth and productivity of many crops and natural vegetation [1–3]. Over the past three decades, O_3_ levels have continued to rise at a rate of 0.5–2.0% per year over the mid-latitudes of the Northern Hemisphere [2]. If current emission trends continue, O_3_ concentrations in the Northern Hemisphere are projected to increase further by 20–25% between 2015 and 2050, and by 40–60% by 2100 [4]. Meanwhile, ultraviolet radiation (UV; 280–400 nm) represents a relatively small but important part of the solar spectrum for higher plants. Exposure to UV, especially the shorter wavelengths in the UV-B region (280–315 nm), has the potential to exert a number of deleterious effects on plants and crops, including the disruption of the integrity and function of biological macromolecules (DNA, proteins and lipids), oxidative damage, partial inhibition of photosynthesis, and ultimately a reduction in growth and productivity [5, 6]. Over the past three decades, UV-B reaching the Earth’s surface has increased by approximately 5% over northern mid-latitudes due to stratospheric O_3_ depletion and is expected to continue to increase until the mid-21^st^ century [7]. Considering the coexistence of high levels of tropospheric O_3_ concentrations and UV radiation, it is essential to study their combined effects on the productivity and quality of important agricultural crops and natural vegetation [8].

Previous studies have shown that high levels of tropospheric O_3_ damaged most crop species and could significantly reduce food production in the future [9, 10]. Indeed a conservative assessment of the crop yield loss due to elevated O_3_ estimates a decrease of 2–16% for wheat, rice and corn and 28–35% for soybeans in China, Japan and South Korea in 2020 [11]. Enhanced UV radiation also causes a reduction of growth and biomass in many plant species [5, 12, 13]. However, most of our knowledge is still limited to the individual effects of O_3_ and UV radiation on crop yields and plant growth. There are few studies conducted to date concerning the combined effects of O_3_ and UV radiation, none of which reached clear conclusions [14–18].

Previous studies showed that changes in the quality (i.e., phenol, flavonoids, lipids, starch, fatty acids) of crops and plants might be an important reason for the adverse effects of O_3_ or UV radiation on crop yields and plant growth [19–21]. However, studies regarding the combined stresses of O_3_ and UV radiation on secondary metabolites of the important agricultural crops and economically important plants are still scarce. Tripathi et al. [22] found that the combined treatment with UV-B + O_3_ induced an increase in phenol content, but the increment was less than that when treated with individual stressors. Ambasht and Agrawal [14] also reported increases of phenol in wheat under the combined treatment. Ormrod et al. [23] found that the levels of flavonoids on a leaf fresh weight basis increased substantially in response to short-term (48 h) UV-B radiation, and exposure to O_3_ before or after UV-B treatment did not consistently affect the levels of these UV-absorptive compounds. Furthermore, hormones are considered to be a primary component of the signaling pathways that control cell division, cell elongation and protein synthesis within apical meristems. Hormonal changes not only influence the adaptive response to environmental changes but also affect normal growth and development [24]. Meanwhile, environmental signals can modulate a plant’s responses to environmental stress through changes not only in hormone concentrations but also in ratios [25]. Hence, it is necessary to study how the increased O_3_ and/or UV radiation change the concentrations and ratios of hormones. Unfortunately, the levels of various hormones in the growth of crops and plants in environments with increased O_3_ and/or UV radiation remain largely unknown.

Soybeans (*Glycine max*) are one of the most important crops in the world. With the rapid increase in O_3_ and UV radiation as a result of industrialization and anthropogenic activities, it is essential to study the effect of elevated O_3_ and/or enhanced UV radiation on soybean yield. The effects of elevated O_3_ or enhanced UV radiation on the growth, morphology and yield of soybeans have been studied widely [26–28]. However, the effects of high levels of O_3_ and/or UV radiation on secondary metabolites and endogenous hormones of soybean leaves have not been investigated, and little information is available concerning the effects of high levels of O_3_ and/or UV radiation on the relationship among secondary metabolites, endogenous hormones and soybean yields. Since the changes in secondary metabolites and endogenous hormones of soybeans might be the mechanism for the severe impact of high levels of O_3_ and/or UV radiation on crop yields, the objective of this study was to examine how high levels of O_3_ and/or UV radiation affect secondary metabolites and endogenous hormones of soybean leaves as well as the seed yield per plant, using open top chambers (OTC). Meanwhile, soybeans might have naturally high levels of floral and pod loss, and subsequent yield loss is greatest when stress occurs during flowering and early pod development [29]. Therefore, the branching, flowering and podding stages of soybeans were chosen to evaluate the temporal variations in secondary metabolites and endogenous hormones under the treatments of elevated O_3_ and/or enhanced UV radiation. The hypothesis of this study is that the combined effect of elevated O_3_ and enhanced UV radiation on the seed yield per plant was more detrimental than the individual effects due to the changes in secondary metabolites and endogenous hormones.

## Materials and methods

### Experimental site and design

The experimental site is located in the Shenyang Experimental Station of Ecology, Chinese Academy of Sciences (41°31′ N, 123°22′ E). This region has a continental monsoon climate with a mean annual temperature of 7.0–8.0°C, annual precipitation of 650–700 mm, and an annual frost-free period of 147–164 days. The soil (0–15 cm) at the study site is classified as an aquic brown soil (silty loam Hapli-Udic Cambosols in Chinese Soil Taxonomy), with 11.28 g kg^-1^ organic C, 1.20 g kg^-1^ total N, 0.41 g kg^-1^ total P, pH (H_2_O) 6.7 at the 0–15 cm depth.

The study was conducted on soybean plants grown in open-top chambers (OTCs), which were established in 2008. The OTCs were 1.15 m in diameter and 2.4 m in height, with a 45° sloping frustum, and the minimum distance between any two chambers was 4 m. The potted soybean cultivar was Tiefeng 29, which was seeded in each pot (26 cm × 36 cm) on May 20, 2015. Soil in the 0–15 cm layer was collected at the study site and was mixed thoroughly after removing roots and organic residues. After sieving (2 mm), the soils were used in the pots of soybean cultivar. NH_4_H_2_PO_4_ at 300 kg hm^2^ was applied to all experimental plots before sowing. The plants were irrigated daily to avoid water stress and appropriate measures were taken to keep the plants free from any biotic, disease or grass stresses. Five plants in the three-leaf stage were established in each pot, and the pots were moved into the OTCs for ozone fumigation. Each OTC was divided into 3 subplots; thus, there were a total of 12 pots in each OTC: 4 collection periods (branching stage, flowering stage, podding stage and the final harvest stage) with 3 subplots (replications). Plants were exposed to elevated O_3_ or/and UV radiations for 8 h (09:00–17:00) per day in the middle of the photoperiod from June 20 to August 12. Expanding leaves with the same leaf age, used for analysis of secondary metabolites and endogenous hormones, were only collected from the top position on the main stem. The soybean leaves were immediately frozen in liquid nitrogen and stored at -70°C until further analysis. The leaves from each pot in each OTC were analyzed independently (for MDA, flavonoids, hormones, etc.), and the results were averaged to calculate a chamber mean for statistics. Seed weight parameters were measured at the time of the final harvest at the end of September using 9 plants from each treatment. The number and weight of seeds per plant and the weight of 100 seeds were calculated. Leaf samples were collected at the branching stage (June 30, 2015), flowering stage (July 24, 2015) and podding stage (August 12, 2015).

The experimental design was based on completely randomized plots that included four treatments: (1) control (hereinafter referred to as CK, ambient O_3_ concentration of approximately 45 nmol mol^-1^; ambient UV radiation intensity of approximately 25.92 kJ h^-1^ m^-2^); (2) elevated O_3_ (O_3_ concentration of 110 ± 10 nmol mol^-1^; no artificial UV tube); (3) UV (ambient O_3_ concentration of approximately 45 nmol mol^-1^; UV radiation intensity of ambient + 1.73 kJ h^-1^ m^-2^); (4) O_3_ + UV (a combination of elevated O_3_ (110 ± 10 nmol mol^-1^) and UV (ambient + 1.73 kJ h^-1^ m^-2^)). Each treatment had 3 replicated OTCs, so in total there were 12 OTCs (3 OTCs × 4 treatments) in our study. Each OTC was divided into 3 compartments that were subjected to the same treatment in order to reduce variability within the same chamber; thus, there were 3 replications (3 OTCs) for each treatment.

O_3_ was produced from pure oxygen with an O_3_ generator (GP-5J, China). O_3_ concentrations were continuously monitored by O_3_ analyzers (S-900 Aeroqual, New Zealand) and were controlled by computers using a software program for O_3_ dispensing and monitoring [30].

UV radiation was artificially supplied by 40 W narrow-band fluorescent tubes (peak value was 305 nm, Beijing Lighting Research Institute) held in mobile and adjustable frames over each pot row. In UV treatments, UV tubes were covered with 0.08 mm cellulose diacetate filters (to absorb radiation below 280 nm). The spectrum of these lamps largely falls into the UV-B band, with a very small amount of UV-A radiation and blue light; thus, in the present study, UV radiation contained UV-B and UV-A. The distance between the top canopies of the plants and the lamps was maintained at 40 ± 2 cm by the mobile frames to provide UV doses of 1.73 kJ h^-1^ m^-2^, equivalent to a 5% increase, on average, of ambient UV radiation (25.92 kJ h^-1^ m^-2^) in Shenyang during clear sky conditions in the summer from 09:00–17:00. UV radiation was monitored by UV radiometer (UV 340B, China).

### Leaf analyses

MDA was measured as thiobarbituric acid-reactive material from centrifuged leaf extracts in 10% trichloroacetic acid [31]. Soybean leaves (500 mg) were ground into a fine powder and then were homogenized in trichloroacetic acid (TCA). After centrifugation, the supernatants were mixed with 0.5% thiobarbituric acid (TBA). The mixture was incubated at 95°C for 30 min, and the reaction was stopped by placing the mixture on ice for 5 min. After centrifugation, the absorbance of the supernatant was measured at 532 nm and 600 nm. After subtracting non-specific absorbance (600 nm), the MDA concentration was determined by its extinction coefficient of 155 mM^-1^ cm^-1^ and expressed as μmol g^-1^ of fresh weight.

Fresh leaves (200 mg) were washed with ion-free water and were cut into tubes with 20 mL of the ionized water. After shaking for 30 min, a DDS-11A Type conductivity meter was used to determine the conductivity as *E*_1_. The solutions were then incubated in boiling water bath for 10 min, and the total conductivity *E*_2_ was determined after cooling. Conductivity in ion-free water was denoted as *E*_0_. The relative electrical conductivity (*R*) was calculated by the formula: *R* = [(*E*_1_−*E*_0_)/(*E*_2_−*E*_0_)]×100%.

The total flavonoid concentrations were determined by a modified method of Chen et al. [32] and Geissman [33]. The total polyphenol concentration was determined by the Folin-Ciocalteu method [34]. An Agilent (Waldbronn, Germany) 1100 HPLC series, which consists of a degasser, binary pump, auto-sampler, thermostat, and photodiode array detector, with a C18-column (Hypersil ODS, 250 mm × 4.6 mm) was used to determine the concentrations of morin, quercetin, ferulic acid and P-coumaric [35, 36]. Pure compounds of morin, quercetin dehydrate, ferulic acid and P-coumaric (Sigma, China) were used as external standards.

Extraction, purification, and determination of endogenous levels of IAA, ABA and ZR were measured by an indirect ELISA technique, as described by Teng et al. [37]. The frozen samples (1 g) were ground under liquid nitrogen, extracted with ice-cold 80% methanol (v/v) containing 1 mmol L^-1^ butylated hydroxytoluene to prevent oxidation, and then stored overnight at 4°C for 16 h in the dark. After centrifugation at 4°C, the supernatants were passed through a C18 Sep-Pak cartridge (Waters, Milford, MA, USA). The efflux was collected and dried in N_2_, and dissolved in a 0.01 mol L^-1^ phosphate buffer solution (pH 7.4), and the concentrations of IAA, GA, ZR, and ABA were determined in an enzyme-linked immuno-sorbent assay (ELISA) using methods described in a previous publication [38].

### Statistical analysis

The differences in the seed yield per soybean plant, the MDA concentration of soybean leaves, relative electrical conductivity of soybean leaves, secondary metabolites concentrations in soybean leaves and endogenous hormone concentrations in soybean leaves in the four treatments of CK, O_3_, UV radiation and O_3_ + UV were evaluated by a one-way analysis of variance (ANOVA) (SPSS 16.0). Multiple comparisons among chamber means (*n* = 3) of the seed yield per plant, MDA concentration, relative electrical conductivity, secondary metabolites concentrations and endogenous hormone concentrations were performed with Tukey’s multiple comparisons test.

The characteristics of secondary metabolites and endogenous hormones were standardized (using the “standardize species” option) before an unconstrained principal component analysis (PCA) (Canoco 5.0). We used PC scores, rather than concentrations of the characteristics of secondary metabolites and endogenous hormones themselves, because some characteristics of secondary metabolites and endogenous hormones co-varied with each other and were not statistically independent. Linear regressions between seed yield per plant and PC scores of characteristics of secondary metabolites and endogenous hormones were used to determine the effects of the characteristics of secondary metabolites and endogenous hormones on seed yield per plant (OriginPro 9.0). Significance was evaluated at *α* = 0.05 in all cases.

## Results

### The seed yield of soybean

Seed number and seed yield per soybean plant were significantly higher under the CK treatment than under the other three treatments (Table 1). There were no significant differences in seed number and seed yield per soybean plant between the elevated O_3_ treatment and enhanced UV treatment groups. Seed number and seed yield per soybean plant under the O_3_ + UV treatment were significantly lower than under the other three treatments.

**Table 1:** The seed yield per soybean plant (±SE, *n* = 3) under stresses of elevated O_3_ and enhanced UV radiation.

Treatments	Seed number per plant	100-seed weight (g)	Seed yield per plant (g)
CK	65.3(7.5)a	25.6(3.5)a	10.8(4.1)a
O_3_	40.3(0.7)b	15.2(2.4)b	5.3(0.1)b
UV	44.4(6.0)b	19.4(5.1)b	7.0(1.7)b
O_3_+UV	34.9(0.9)c	10.4(1.4)c	3.4(1.3)c

Different letters in columns indicate statistical difference among the four treatments according to Tukey’s test (P < 0.05).

### MDA and relative electrical conductivity of soybean

**Fig 1:**
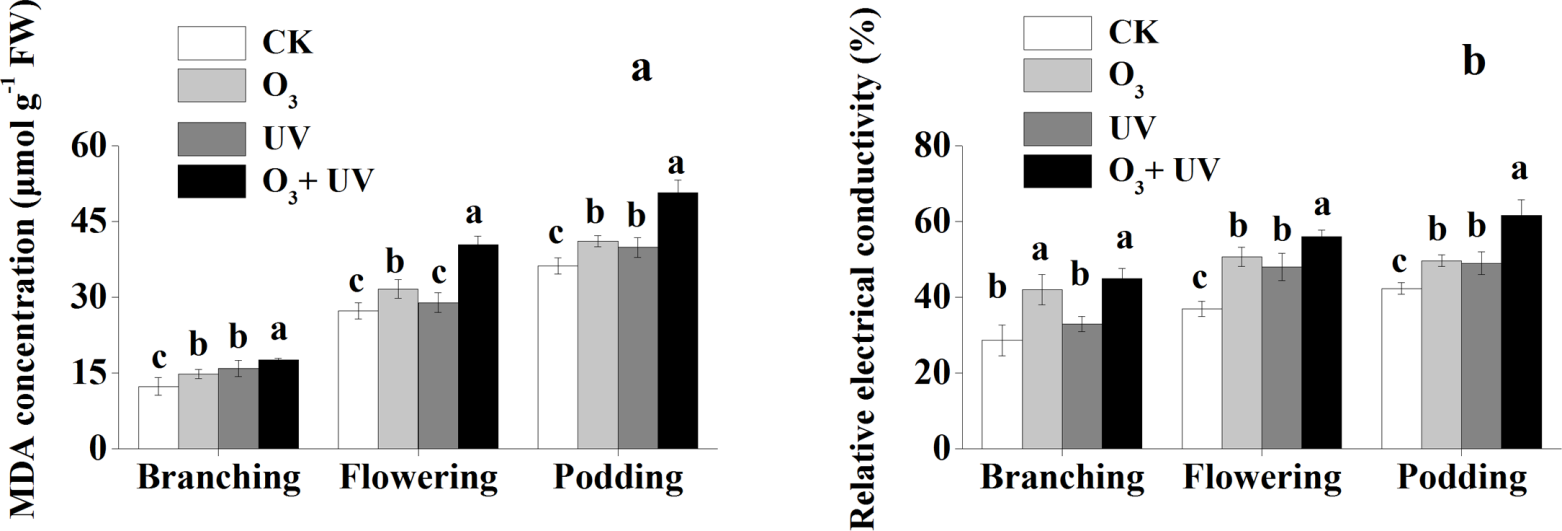
MDA concentration (μmol g^-1^ FW) (a) and relative electrical conductivity (%) (b) of soybean leaves under elevated O_3_ and UV radiation at branching, flowering and podding stages. Data are means ± SE, with *n* = 3 for each treatment. Different letters above the bars represent significant differences from Tukey’s multiple comparisons among four treatments (*P*<0.05).

The MDA concentration of soybean leaves was significantly lower under CK treatment than under the other three treatments at the branching stage (Fig 1A). There was no significant difference in the MDA concentration of soybean leaves between the CK treatment and the enhanced UV treatment at the flowering stage. The MDA concentration was significantly higher under the O_3_ + UV treatment than under the other three treatments at the flowering and podding stages. There was no significant difference in the MDA concentration of soybean between the elevated O_3_ treatment and the UV radiation treatment at the podding stage.

The relative electrical conductivity of soybeans under the elevated O_3_ treatment and the O_3_ + UV treatment was significantly higher than that under the other two treatments at the branching stage (Fig 1B). The relative electrical conductivity of soybeans under the CK treatment was significantly lower than that under the other three treatments at the flowering and podding stages. The relative electrical conductivity of soybeans under the O_3_ + UV treatment was significantly higher than that under the other three treatments at the flowering and podding stages. There was no significant difference in the relative electrical conductivity of soybeans between the elevated O_3_ treatment and the UV radiation treatment at the flowering and podding stages.

### Secondary metabolites and endogenous hormones of soybean leaves

**Fig 2:**
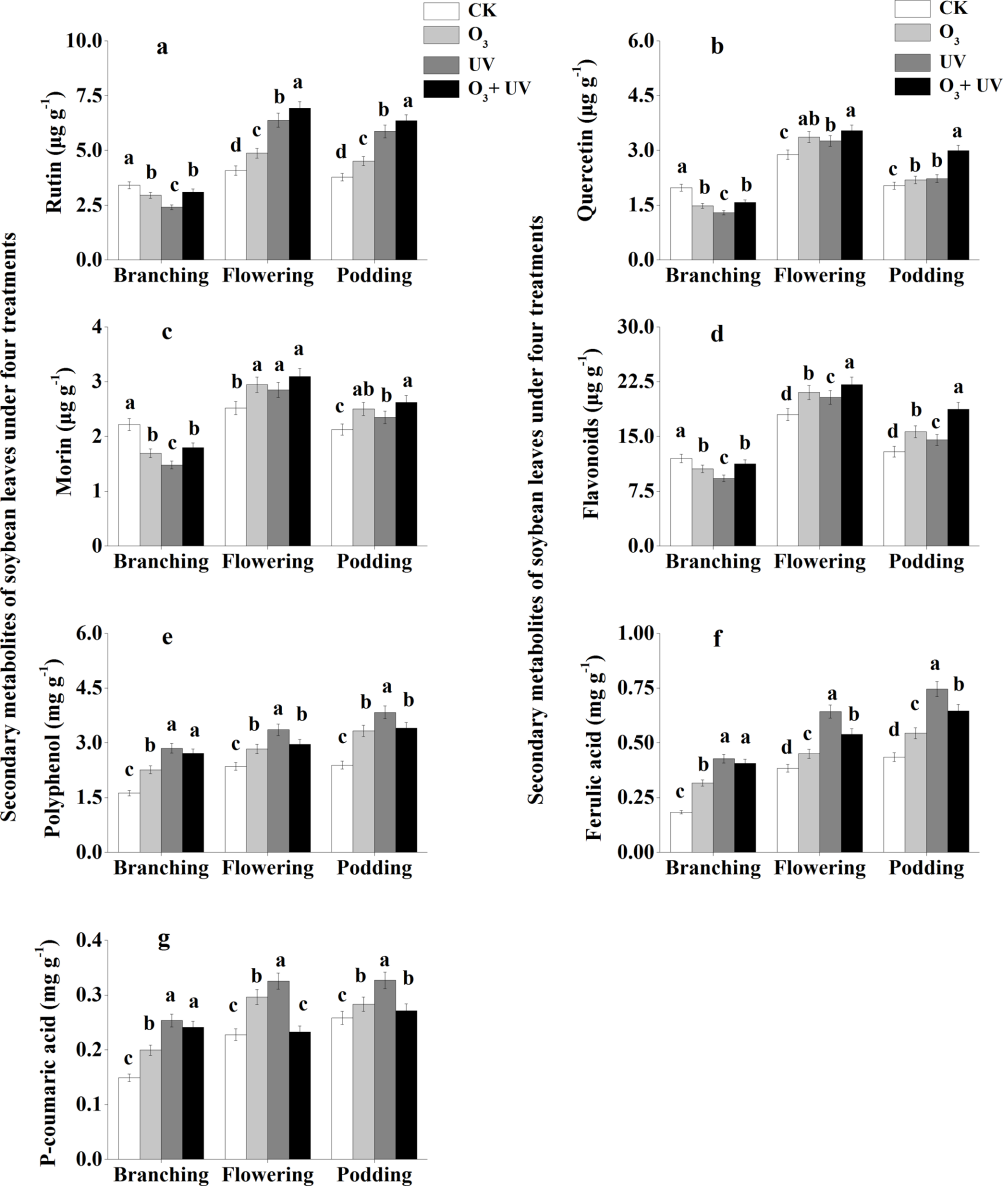
Secondary metabolites of soybean leaves under elevated O_3_ and UV radiation at branching, flowering and podding stages. Data are means ± SE, with *n* = 3 for each treatment. Different letters above the bars represent significant differences from Tukey’s multiple comparisons among four treatments (*P*<0.05).

Soybean leaves had significantly higher concentrations of rutin, quercetin, morin and flavonoids under the CK treatment than under the other three treatments at the branching stage (Fig 2). Soybean leaves had significantly lower concentrations of rutin, quercetin, morin and flavonoids under the CK treatment than under the other three treatments at the flowering and podding stages. Polyphenol and ferulic acid concentrations of soybean leaves were significantly lower under the CK treatment than under the other three treatments at the branching, flowering and podding stages (Fig 2E and 2F). The P-coumaric concentration of soybean leaves under the CK treatment was significantly lower than that under the other three treatments at the branching and podding stages (Fig 2G).

**Fig 3:**
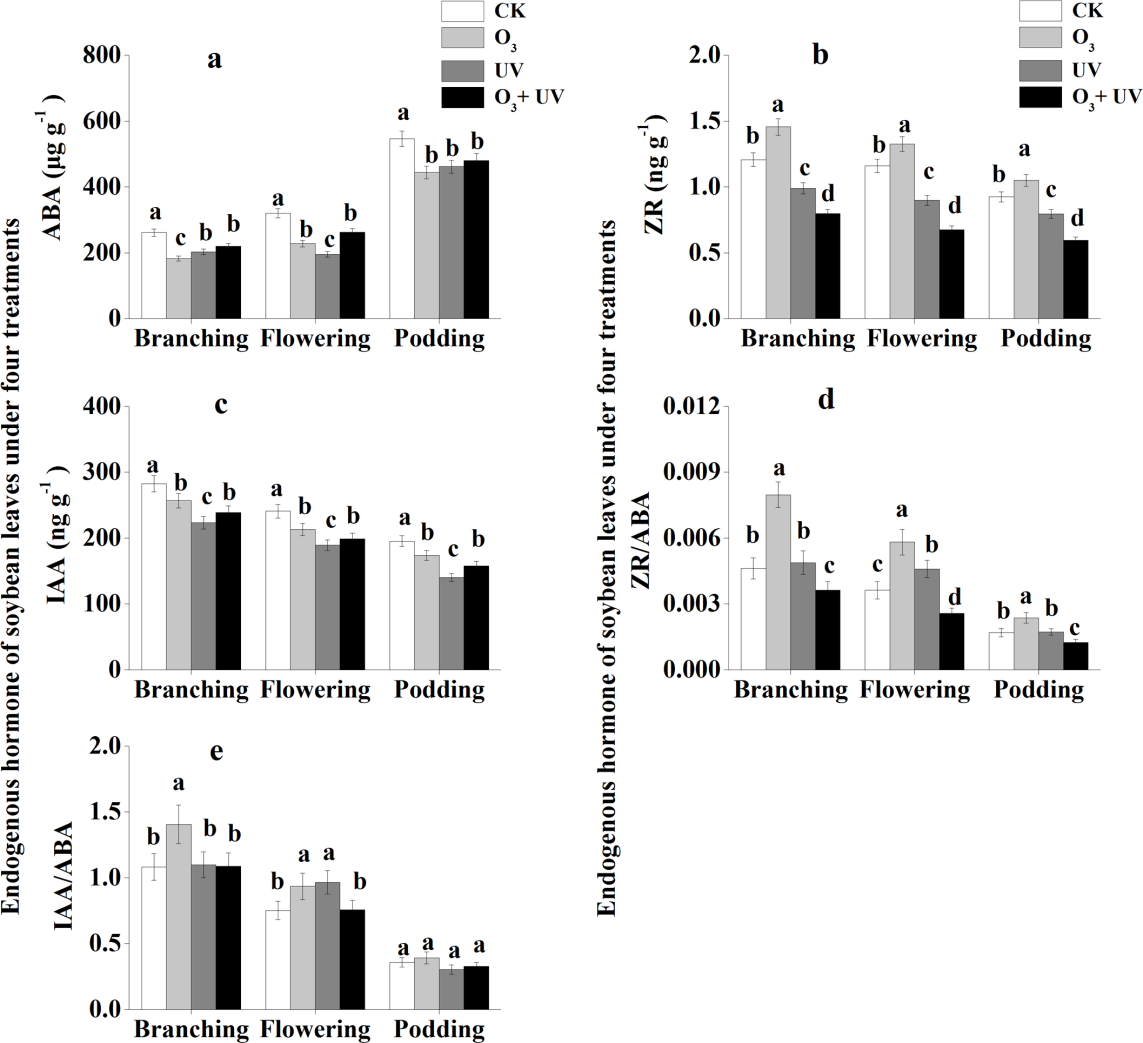
Endogenous hormones of soybean leaves under elevated O_3_ and UV radiation at branching, flowering and podding stages. Data are means ± SE, with *n* = 3 for each treatment. Different letters above the bars represent significant differences from Tukey’s multiple comparisons among four treatments (*P*<0.05).

Concentrations of ABA and IAA in soybean leaves under the CK treatment were significantly higher than those under the other three treatments at the branching, flowering and podding stages (Fig 3A and 3C). The ZR concentration of soybean leaves under the CK treatment was significantly lower than that under elevated O_3_ treatments and significantly higher than those under the treatments of UV radiation and O_3_ + UV at the branching, flowering and podding stages (Fig 3B). There was no significant difference in the ZR/ABA ratio of soybean leaves between the CK treatment and the UV radiation treatment at the branching and podding stages (Fig 3D). The ZR/ABA ratio of soybean leaves under the O_3_ treatment was significantly higher than that under the other three treatments at the branching, flowering and podding stages. There were no significant differences in the IAA/ABA ratio of soybean leaves between the CK treatment, UV radiation treatment and O_3_ + UV treatment at the branching stage (Fig 3E). The IAA/ABA ratio of soybean leaves under the CK treatment was significantly lower than that under the elevated O_3_ treatment and UV radiation treatment at the flowering stage. There were no significant differences in the IAA/ABA ratio of soybean leaves among the four treatments at the podding stage.

**Fig 4:**
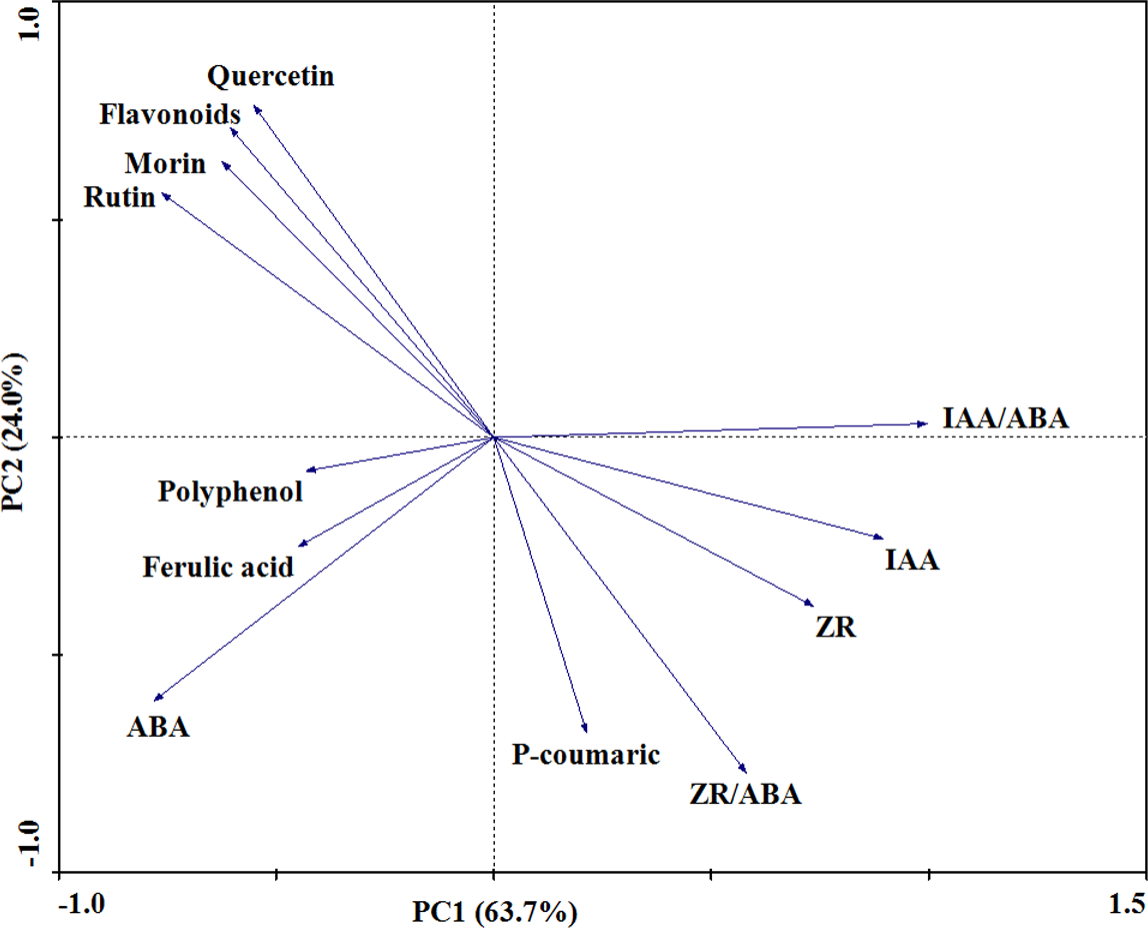
Principal component analysis of secondary metabolites and endogenous hormones of soybean leaves under elevated O_3_ and UV radiation. The first two principal components (PCs) accounted for 63.7% (PC1) and 24.0% (PC2) of the total variation.

**Fig 5:**
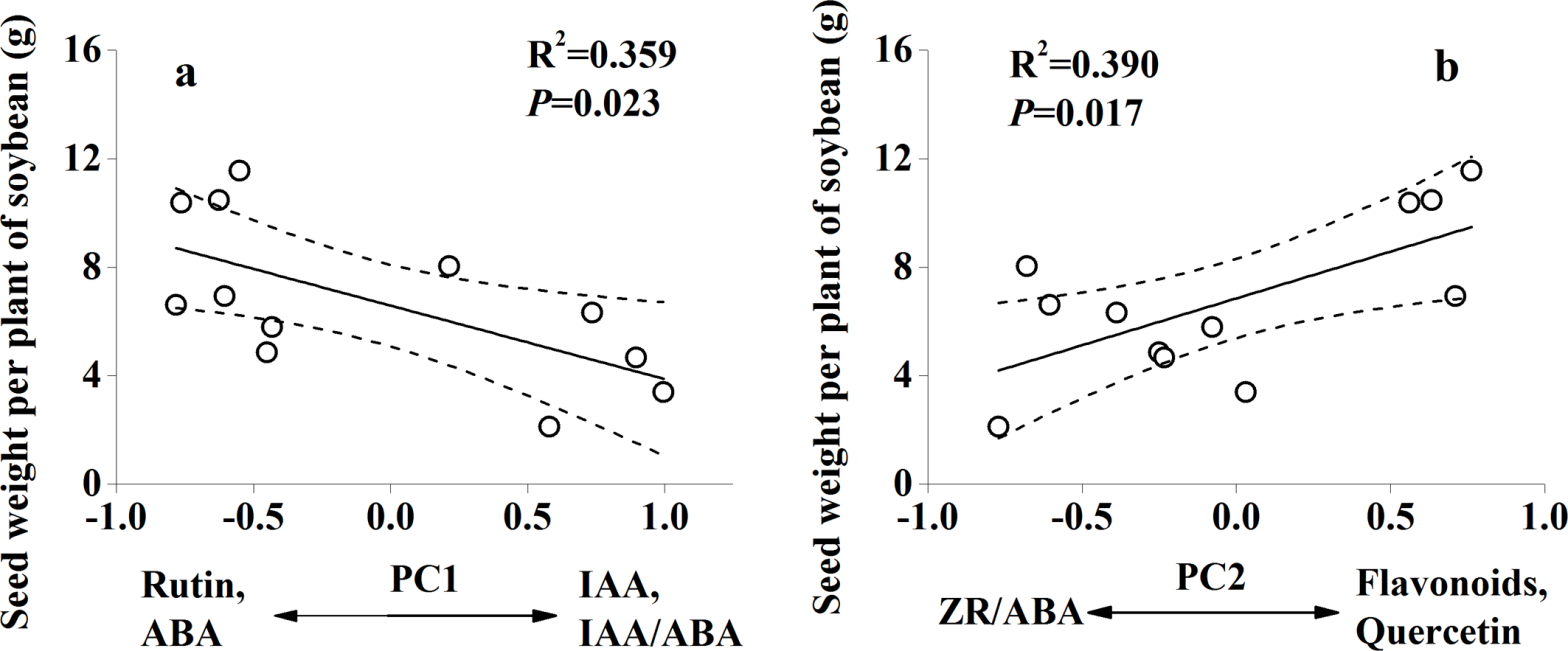
Seed yield per soybean plant as a function of the first PC (a) and second PC (b). Dashed lines represent the 95% confidence interval of the regression.

The PCA analysis showed that the secondary metabolites and endogenous hormones of soybean leaves were clearly separated (Fig 4). PC1and PC2 together explained 87.7% of the variation in the secondary metabolites and endogenous hormones of soybean leaves. Seed yield per plant had a significant association with PC1 and PC2 scores, while seed yield per plant had no significant association with PC3 scores (*P* = 0.023, *P* = 0.017 respectively; Fig 5).

## Discussion

The major objective of the present study was to assess the impact of elevated O_3_ and enhanced UV radiation individually as well as in combination on the seed yield per soybean plant, secondary metabolites and endogenous hormones of soybean leaves. Our results confirmed previous findings in which elevated O_3_ and enhanced UV radiation individually decreased seed yield per plant [11, 13]. Liu et al. [39] found that the yields of three soybean cultivars were decreased by 43.7% by UV radiation. The seed yield per soybean plant decreased by 35.2% and 50.9% under high levels of UV radiation and O_3_, respectively, in the present study. Furthermore, the combination of elevated O_3_ and enhanced UV radiation decreased the seed yield per soybean plant by 68.5%, indicating that the effect of combined stress of elevated O_3_ and enhanced UV radiation on seed yield per soybean plant was greater than the effects of individual stresses, which was consistent with the hypothesis that the combined stress of increased O_3_ and UV radiation was more detrimental than individual stresses in the present study. Our results are different from a previous study by Miller et al. [15], who found that UV + O_3_ treatment did not have a significant effect on soybean yield, but the individual response to O_3_ was significant.

In addition, O_3_ is well known to affect the function of plasma by disorganizing the membrane structure and altering membrane permeability through lipid peroxidation and electrolyte leakage [40, 41]. In the present study, the MDA concentration and relative electrical conductivity were drastically enhanced by elevated O_3_, indicating that O_3_ intensified the accumulation of reactive oxygen species (ROS) induced by oxidative stress and the degree of lipid peroxidation of the leaf tissue membrane [42, 43]. Similarly, Rai and Agrawal [44] also reported increased lipid peroxidation in rice plants after O_3_ exposure. Meanwhile, the O_3_ + UV treatment resulted in a higher MDA concentration and relative electrical conductivity than the elevated O_3_ treatment or enhanced UV radiation treatment alone, indicating that supplemental O_3_ aggravated the oxidative stress of UV radiation. In contrast, Tripathi et al. [22] showed that the combined stress of O_3_ and UV radiation effected membrane lipids was less compared to their individual effects.

The stress of enhanced UV radiation and elevated O_3_ individually not only significantly decreased the seed yield per plant but also changed the concentration of secondary metabolites in the present study. Flavonoids are produced as protective substances against UV radiation in plants [21]. Some studies have shown that flavonoids, as an effective abiotic elicitor, are highly sensitive to UV radiation and their concentrations usually correlate positively with UV [45, 46]. Rutin (sometimes called vitamin P) displays strong antioxidant activity, which could alleviate the damage from UV stress. Tsurunaga et al. [47] found that the rutin content of buckwheat sprouts was enhanced under various levels of UV radiation. Huang et al. [36] showed that the content of rutin and quercetin of hairy roots and all parts of tartary buckwheat were increased under UV stress. Similarly, in the present study, the concentrations of total flavonoids, rutin and quercetin under the stress of elevated O_3_, enhanced UV radiation and O_3_ + UV increased at the flowering and podding stages, while at the branching stage, the concentrations of total flavonoids, rutin and quercetin under the three treatments decreased. These results suggest that the changes of concentrations of total flavonoids, rutin and quercetin under the three treatments depended on leaf stage, similar to the findings of Kolb et al. [48], Reifenrath and Müller [49], Londoño et al. [50] and Kuhlmann and Müller [46], mainly because the capacity for the formation of secondary metabolites in the epidermis is highly leaf-age dependent [51].

Furthermore, the concentrations of rutin, quercetin and total flavonoids showed significant positive correlations with seed yield per soybean plant in the present study, while polyphenol, ferulic acid and P-coumaric had insignificant correlations with seed yield per soybean plant. The biosynthesis of many secondary metabolites in plants is usually considered a common defense response of plants to biotic and abiotic stresses, and their accumulation could be stimulated by biotic and abiotic elicitors [52]. Therefore, rutin, quercetin and total flavonoids, rather than polyphenol, ferulic acid and P-coumaric, might have important regulatory roles in the decrease of seed yield per plant under the stress of elevated O_3_, enhanced UV radiation and O_3_ + UV, which was consistent with the hypothesis of the present study.

The leaf ABA concentration decreased under high levels of O_3_ and UV radiation and was significantly positively correlated with seed yield per plant. Similarly, Li et al. [53] found that the ABA concentration of needles of Chinese pine decreased under elevated O_3_. ABA has been identified as a messenger in stress–perception–response pathways, and the stress may be drought, cold, salinity stress or air pollution [54, 55]. Several studies have demonstrated the effects of ABA on the abundance of many mRNAs and proteins, particularly detoxification proteins, but the mechanisms by which ABA-induced stress proteins lead to stress tolerance remain unknown [56]. An important role of endogenous ABA is to limit ethylene production, and as a result, ABA may often function to maintain rather than inhibit shoot and root growth [57].

IAA is the predominant auxin in most plants, with higher levels in young, growing tissues [58]. In the present study, elevated O_3_ and enhanced UV radiation decreased the IAA concentration, similar to the findings of Li et al. [25]. Meanwhile, the present study showed that the IAA concentration was significantly negatively correlated with the seed yield of soybeans, in contrary to the findings of Bartel [58]. Furthermore, the ratios of ZR/ABA and IAA/ABA also showed significant negative correlations with the seed yield of soybeans. Thus, the distribution of photoassimilate within the plants may be influenced not only by levels of a specific hormone but also by its interactions with other hormones [59].

In addition, in the present study, both stresses had negative effects on the seed yield of soybeans, but the magnitude of their individual effects was always lower than that of their combined effect, indicating that the combined stress induces more damage compared to the individual stresses. It might be possible that UV radiation and O_3_ differ in their action as stressors, although they both lead to the damage of the membrane structure and membrane permeability. There is a general consensus that O_3_ enters mesophyll cells *via* stomata and then degrades in the apoplast, forming O_2_^-^, HO• and H_2_O_2_. Stomatal conductance is one of the determining factors for O_3_ uptake in plants [60]. Previous studies have shown that ABA may have an important role in controlling stomatal response, and it might induce closure of the stomata, which would result in decreased phytotoxicity of O_3_ [61, 62]. In this study, there were no significant differences in ABA concentrations between the elevated O_3_ treatment and O_3_ + UV treatment at the flowering and podding stages, indicating that supplemental UV might not cause changes in the stomatal response to the elevated O_3_ treatment. Meanwhile, UV radiation could activate membrane-localized NADPH oxidase or promote secondary metabolite accumulation in plant cell and tissue cultures, which then leads to the generation of ROS [63, 64]. In our study, the combined stress increased the concentrations of rutin, quercetin and total flavonoids, which showed significant correlation with seed yield per plant, compared to individual stress at the flowering and podding stages. Therefore, supplemental O_3_ might exacerbate the UV damage on soybean leaves. Plants respond differently to treatment with both UV radiation and O_3_.

Notably, Miller et al. [15] found that UV + O_3_ did not have a significant effect on soybean yield. Ambasht and Agrawal [14] reported the induction of oxidative stress in UV and O_3_ provided singly or in combination, and the response of wheat to their combination was always less than the responses to the individual stressors. These different results may be because the ROS might be regulated in a dose-dependent manner and because of cultivar differences [65]. Soybeans are a N_2_-fixing species and may thus be more O_3_-sensitive compared to other crops, such as wheat [14]. Meanwhile, the elevated O_3_ (110 ± 10 nmol mol^-1^ 8 h per day) used in this study was higher than that in other studies; for example, O_3_ treatment concentrations ranged from 14 to 83 nL L^-1^ (mean concentrations treated for 12 h per day in a season) in the study by Miller et al. [15]. The high concentration of O_3_ used in this study showed a significant impact on O_3_-sensitive soybeans, which might lead to more severe effects from a combination of the two stresses on seed yield and flavonoids concentrations, similar to Feder and Shrier [16].

It is worth mentioning that the acquisition of knowledge regarding the understanding of the effects of enhanced UV radiation on secondary metabolites and endogenous hormones of soybean leaves was mainly obtained from the use of UV-B lamp. While most of the UV-B lamp spectrum belongs to the UV-B band, the lamps also have small amount of UV-A radiation and blue light. It has been known that UV-A radiation and blue light are able to penetrate deeper than UV-B radiation into leaves and produce ROS [66]. Several studies have paid attention to the effects of UV-A radiation and blue light on secondary metabolites of plant and crop in recent years [67, 68]. Whether such findings matter merits further investigation.

## Conclusions

The present study showed that elevated O_3_ and enhanced UV radiation individually, as well as in combination highly damaged soybean growth mediated by changes in secondary metabolites and endogenous hormones. The concentrations of total flavonoids, rutin and quercetin under the combined stress of elevated O_3_ and enhanced UV radiation were significantly increased compared to that under individual stresses at the flowering and pooding stages, suggesting that supplemental O_3_ might exacerbate the UV damage on soybean leaves. Flavonoids rather than polyphenols might have an important regulatory role on the decrease of seed yield per plant under the stress of elevated O_3_, enhanced UV radiation and O_3_ + UV. In addition, the combined stress of elevated O_3_ and enhanced UV radiation showed negative effects on seed yield per plant, and the magnitude of their individual effects was always lower than that of their combined effect.

## Abbreviations

MDA: malondialdehyde
ABA: abscisic acid
ZR: zeatin riboside
IAA: indole-3-acetic acid
ROS: reactive oxygen species
